# Automatic Extraction of Muscle Parameters with Attention UNet in Ultrasonography

**DOI:** 10.3390/s22145230

**Published:** 2022-07-13

**Authors:** Sofoklis Katakis, Nikolaos Barotsis, Alexandros Kakotaritis, George Economou, Elias Panagiotopoulos, George Panayiotakis

**Affiliations:** 1 Electronics Laboratory, Department of Physics, University of Patras, 26504 Patras, Greece; kakotaritis.alexandros@gmail.com (A.K.); economou@physics.upatras.gr (G.E.); 2 Department of Medical Physics, School of Medicine, University of Patras, 26504 Patras, Greece; nbarotsis@upatras.gr (N.B.); panayiot@upatras.gr (G.P.); 3 Orthopaedic and Rehabilitation Department, Patras University Hospital, 26504 Patras, Greece; ecpanagi@med.upatras.gr

**Keywords:** ultrasonography, muscle thickness, segmentation, Attention-UNet, pennation angle, fascicles length

## Abstract

Automatically delineating the deep and superficial aponeurosis of the skeletal muscles from ultrasound images is important in many aspects of the clinical routine. In particular, finding muscle parameters, such as thickness, fascicle length or pennation angle, is a time-consuming clinical task requiring both human labour and specialised knowledge. In this study, a multi-step solution for automating these tasks is presented. A process to effortlessly extract the aponeurosis for automatically measuring the muscle thickness has been introduced as a first step. This process consists mainly of three parts. In the first part, the Attention UNet has been incorporated to automatically delineate the boundaries of the studied muscles. Afterwards, a specialised post-processing algorithm was utilised to improve (and correct) the segmentation results. Lastly, the calculation of the muscle thickness was performed. The proposed method has achieved similar to a human-level performance. In particular, the overall discrepancy between the automatic and the manual muscle thickness measurements was equal to 0.4 mm, a significant result that demonstrates the feasibility of automating this task. In the second step of the proposed methodology, the fascicle’s length and pennation angle are extracted through an unsupervised pipeline. Initially, filtering is applied to the ultrasound images to further distinguish the tissues from the other muscle structures. Later, the well-known K-Means algorithm is used to isolate them successfully. As the last step, the dominant angle of the segmented muscle tissues is reported and compared with manual measurements. The proposed pipeline is showing very promising results in the evaluated dataset. Specifically, in the calculation of the pennation angle, the overall discrepancy between the automatic and the manual measurements was less than 2.22° (degrees), once more comparable with the human-level performance. Finally, regarding the fascicle length measurements, the results were divided based on the muscle properties. In the muscles where a large portion (or all) of the fascicles are located between the upper and lower aponeuroses, the proposed pipeline exhibits superb performance; otherwise, overall accuracy deteriorates due to errors caused by the trigonometric approximations needed for the length calculation.

## 1. Introduction

Musculoskeletal ultrasound imaging is an effective modality to assess skeletal muscle’s architectural characteristics and quality. Musical thickness extraction is an important application in clinical routine due to its correlation with specific muscle disorders and its reliability [1] as a measurement. Different studies in the medical literature have thoroughly investigated the relevance of muscle hypertrophy [2], disuse atrophy [3], ageing [4,5] and pathological conditions [6] with muscle thickness. Furthermore, this measurement has been helpful in predicting human subjects’ leg skeletal mass [7] and indirectly evaluating the muscle volume and cross-sectional areas in extremity and trunk muscles [8,9]. More recently, in [10], muscle thickness was utilised for predicting sarcopenia, a common disease in the elderly. Specifically, the muscle thickness of head, neck, upper and lower limb muscles measured with ultrasonography was evaluated as a potential diagnostic tool in sarcopenia. Apart from the muscle thickness, other important muscle parameters in determining muscle performance are the fascicle’s length and its pennation angle [11,12,13]. These measurements have often been used in physiological and biomechanical modelling studies [14,15] to estimate the force-generating capacity of the human muscles.

However, measuring these skeletal muscle parameters requires the visual identification of the deep and superficial aponeuroses from the ultrasound images. The above is a time-consuming and user-dependent task in the clinical routine, thus making it error-prone. Computer-Aided Diagnostic (CAD) systems have recently emerged to facilitate and improve this process. In particular, in studies [16,17], the cross-sectional areas (CSA) extraction of transverse musculoskeletal ultrasound (US) images has been investigated. In [16], aponeuroses are extracted through gradient-based filtering and specialised post-processing, and as a final step, the CSA of the muscle is calculated. Similarly, in [17], the power of deep learning architectures has been incorporated to automatically segment the examined muscles’ CSA. Both studies have produced automated ways to help doctors diagnose musculoskeletal disorders.

Furthermore, at [18], a framework called Muscle Ultrasound Analysis (MUSA) automatically calculates the muscle thickness and pennation angle by extracting the deep and superficial aponeurosis of the muscle with gradient-based filtering proposed. It must be mentioned that this study revealed the feasibility of automatic measurements. However, their approach’s downside is that it is based exclusively on gradient-based filtering, which can be error-prone in low-contrast images.

Another study worth mentioning is [19]. This study utilised a Generative Adversarial Network (GAN) to generate realistic B-mode musculoskeletal ultrasound images. Afterwards, they tested publicly available software to measure the generated images’ muscle thickness and pennation angle. In addition to this, regarding the automatic extraction of the fascicles, in [20], multiscale vessel enhancement filtering was used to preprocess the image. Then, fascicle orientations were quantified using either the Radon transform or wavelet analysis. Our study presents a complete pipeline for automatically extracting the muscle thickness, pennation angle and fascicle length. The state-of-the-art Attention UNet [21] and a customised procedure are incorporated to segment the muscle boundaries automatically. Since the deep and superficial aponeurosis were extracted, the muscle thickness was calculated, and the inner muscle area was isolated. Next, a sophisticated pipeline was utilised to extract the muscle fascicles in this area. As the last step, the fascicles’ length and pennation angle were automatically calculated. 

The main aim of this study is to improve the existing CAD systems by introducing state-of-the-art deep (and machine) learning techniques for a better and more reliable way of calculating important for diagnostic purposes and muscle parameters. Such improvement would be of high value since it will allow ultrasonographers to calculate muscle thickness, pennation angle and fascicles length, faster, more accurately and in a more standardised fashion. Through this attempt, two major contributions are proposed. Firstly, to the best of our knowledge, the Attention UNet have been used for the first time in this type of problem. Similarly, the proposed pipeline to measure the fascicle length and the pennation angle is presented for the first time. Together, these methodologies can lead to an optimised, automated system for assisting the specialised doctor and other healthcare staff as an additional diagnostic tool.

## 2. Materials and Methods

### 2.1. Database

The database used in this study consisted of ultrasound measurements from 74 volunteers (40 males with a mean age of 24.05 ± 3.19 y and 34 females with a mean age of 24.29 ± 3.21 y) also presented in [22,23]. Regarding the ultrasound recordings, all images were acquired using a Logiq P9 ultrasound system (GE Healthcare GmbH, Freiburg, Germany). The ML6-15 linear array transducer was used, operating at 10-MHz frequency. All image optimisation modes were switched off to avoid alteration of image characteristics by software processing, except for harmonic tissue imaging. The gain was set to 50, the dynamic range at 66 dB and both were kept constant throughout the examination of all subjects. The selected muscles and the examination protocol of the recordings that were taken into consideration for evaluating the proposed methodology were:

- The longitudinal ultrasound scans of the Biceps Brachii (BB) muscle at two-thirds of the distance from the acromion to the elbow crease;
- The longitudinal ultrasound scans of the bulkiest part of the medial head of the Gastrocnemius (GCM) muscle;
- The longitudinal ultrasound scans of the Tibialis Anterior (TA) muscle at one-quarter of the distance from the inferior pole of the patella to the malleolus lateralis.

The depth was set at 4 cm for all muscles mentioned above and increased in patients with voluminous muscles to include the whole muscle in the image. The focal zones (up to six) were distributed evenly along with the depth of the image. All measurements were performed in the Rehabilitation Department of the University Hospital of Patras, and this study was conducted according to the Declaration of Helsinki.

After selecting these scans, the data was curated to exclude ill-taken images. As a result, 433 images were included in the evaluated dataset. Of the 433 dataset images, 188 were of the BB muscle, 128 were of the GCM, and the rest 117 were of the TA muscle. For the evaluation process, the well-established hold-out protocol was selected. In particular, for each muscle, the images were randomly split (80–20%) between the training and validation sets.

**Figure 1:**
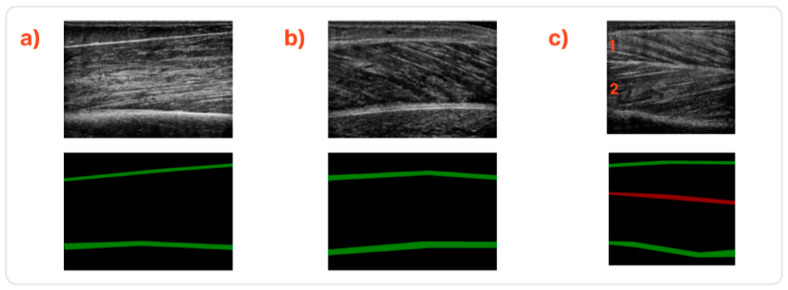
Sample ultrasound images along with their annotations. (**a**) Sample from the BB muscle, (**b**) from the GCM muscle, and (**c**) the TA muscle. The TA muscle comprises two compartments divided by the central fascia in the middle. These are depicted as 1 and 2.

The annotation procedure of the deep and superficial aponeurosis, muscle thickness and pennation angle measurements were performed with the guidelines and overview of a specialised doctor. In Figure 1, samples of the studied muscles are presented along with their annotated counterparts.

### 2.2. Muscle Thickness Calculation

#### 2.2.1. Proposed Method

As the first step of the proposed methodology, the problem of automatically extracting the muscle thickness is investigated. The Attention UNet, an improved variant of the well-established UNet [24], has been incorporated to delineate the superficial and deep aponeuroses. The original version of UNet has shown outstanding performance in biomedical problems [25,26], mostly due to its ability to learn from a small amount of data. The Attention UNet variant is also boosted with attention gates to highlight better salient features passed through the skip connections. According to the authors, these attention gates can filter irrelevant and noisy responses in skip connections of the convolutional network. The benefit of the shallower layers is to be updated primarily based on spatial regions relevant to a given task. 

**Figure 2:**
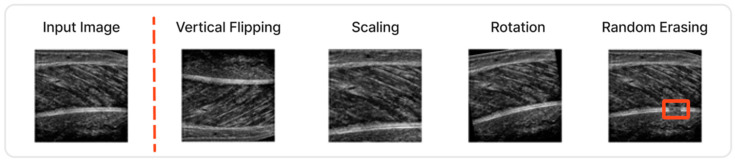
Different image augmentations techniques were utilised during the training of the Attention-UNet.

Image augmentation techniques are another important aspect for optimising the performance of the Attention UNet. Not only are they improving the generalisation ability without the need for extra data, but they also lead to better accuracy results. In our case, several of these techniques were incorporated since the number of available ultrasound images is limited to a few hundred. Specifically, vertical flipping, rotation, scaling and random erasing were some of the applied augmentations. Random erasing, in particular, has been modified to provide more attention to the missing boundaries of the muscles, a common artifact in the field. Some characteristic images are depicted in Figure 2. 

In addition, a weighted dice loss was used for the optimisation process along with an ADAM optimiser [27]. Regarding the learning rate policy, a stepwise decrease of the learning rate was found best fitted. The images were resized to be 256 × 256 to be given as input in the Attention UNet. Finally, the number of epochs was selected to be 300, with the batch size equal to 8.

Once the aponeuroses were identified through Attention UNet, a post-processing refinement was induced. The post-processing pipeline described in Algorithm 1 below was found necessary since, in some images, the Attention UNet could not localise the upper and lower boundary with high precision. These irregularities are associated mainly with two cases. In the first case, the muscle boundaries have gaps across their structure, and in the second, the boundary is composed of a small number of pixels.
**Algorithm 1.** Post-Processing Algorithm**Require:** Prediction array ***x*****Input:**$\mathrm{input}\;\mathrm{array}\;x\in R^{nxn}:\;\left[0-255\right]\;\;$**Output:**$\mathrm{output}\;\mathrm{array}\;\hat{x}\in R^{nxn}:\;\left[0-255\right]\;\;\;$1:**Extract** structures **S = [s_1_, s_2_, …, s_n_]** with **CCA**2:$\;\hat{S}=argmax\left(area\left(S\right)\;,\;2\right)$3:$\hat{H}=S-\hat{S}$4:$\hat{x}=zeros\left(width\left(x\right),\;height\left(x\right)\right)$5:For i=1,26:   **If** (***xmax*(**$\hat{S}$**(*i*)) −**
***xmin*(**$\hat{S}$**(*i*)) == *width*(x))**7:      *//Aponeuroses has no empty spaces*
8:      $\hat{\;x}+=\hat{S}\left(i\right)$
9:   **Else**:10:      $For\;j=1,2,\ldots ,size\left(\hat{H}\right)$
11:         **If**  $\hat{H}\left(j\right)$
***along x_-axis_ of***
$\hat{S}\left(i\right)$12:            $h_{1}\;\;\;=skeletonise\left(\hat{H}\left(j\right)\right)$
13:            $s_{1}\;\;\;\;\;=skeletonise(\hat{S}$) 14:            
$\hat{S}\left(i\right)=polyfit\left(s_{1},\;h_{1}\right)$
15:         **Else**:16:            ***pass***17:         $\hat{\;x}+=\hat{S}\left(i\right)$


The post-processing algorithm takes the prediction mask of the Attention-UNet as input. Initially, the connected structures of this mask are extracted with Connected Component Analysis (CCA). Next, these structures are divided into two subsets. The first subset contains the two biggest structures in terms of area: the aponeuroses candidates, and the second includes all the rest.

For each aponeurosis candidate, we examined if a smaller structure exists along the x-axis. If not, then the aponeurosis is complete. Else, an iteration of all the structures in the second subset then takes place. If a structure is along the x-axis of the aponeuroses candidate, the following actions are performed:

- Skeletonise the aponeuroses candidate;
- Skeletonise the structure;
- Connect these two structures using the polynomial fitting of rank two to fill in the missing pixels (and smoothen the boundaries of possible spikes).

This iterative process stops when there are no other candidates on the x-axis of the aponeuroses candidates. The final output of this process is a new mask cleaned from artifacts and with the deep and superficial aponeuroses filled.

**Figure 3:**
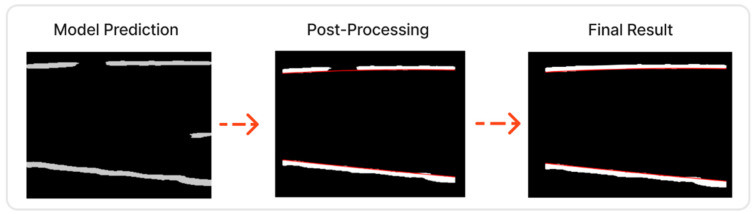
In the case that the predicted mask is not optimal, the post-processing improved the result.

The previously described preprocessing and refinement methodology facilitates the muscle thickness measurement. A characteristic example is shown in Figure 3.

#### 2.2.2. Muscle Thickness Measurements

We follow some modifications and improvements to the basic pipeline procedure described in [18] (MUSA) to measure the muscle thickness. It is known that the muscle thickness in the longitudinal plane is defined as the distance between the superficial and deep aponeuroses. Still, due to its variability along the longitudinal axis, it can be better approximated by cutting the muscle into a few sectors, computing each sector’s distance and taking their mean value as output. 

**Figure 4:**
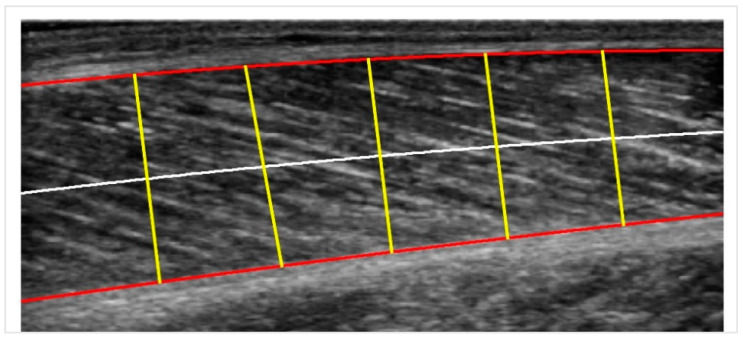
Centerline Distance: the red curves are the two boundaries; the white line is the centerline, and the yellow lines are the perpendicular chords to the centerline used for calculating the muscle thickness.

In particular, a centerline is drawn midway between the superficial and the deep aponeuroses. Then, for each point of the centerline, a chord perpendicular to it is plotted. The length of this chord is the measure of muscle thickness at that point. The average distance for all the chords along the centerline is the centerline distance. The number of sectors is chosen to be five, and the procedure is illustrated in Figure 4. In the last step, the pixels are transformed into physical units (mm) by multiplying by a scale factor retrieved from the DICOM metadata provided by the ultrasound machine. 

### 2.3. Pennation Angle and Fascicles Length Calculation 

#### 2.3.1. Muscle Fascicles Extraction

Once the inner muscle section has been extracted, the next step of the proposed methodology is the segmentation of the muscle fascicles in a simple and automated way. However, ultrasound images are noisy and blurred [28]; thus, certain carefully selected preprocessing steps are needed to achieve this goal. In total, to automatically extract the muscle fascicles:

(a) Firstly, the contrast-limited adaptive histogram equalisation method (CLAHE) [29] enhances the contrast between the fascicles and the other muscle structures.
(b) Subsequently, to highlight linear structures, a filtering approach is applied [30] that is suitable for ridge-like elongated structures reminiscent of the fascicle’s appearance.
(c) The well-established k-means algorithm [31] has been utilised in the next step to delineate the inner fascicles.
(d) Later, the smallest structures (in terms of the area of pixels) are eliminated.
(e) Subsequently, each structure is skeletonised, and the points of the skeleton are fitted to a linear segment. To accomplish continuity of fascicles, the segments that are part of the same line were identified and connected based on the criteria mentioned above.
(f) Afterwards, a selection of the dominant orientation is performed via k-means clustering. In particular, the orientation with the highest total line segment length is chosen from the clusters.
(g) Finally, the line segments are extended to fit the whole muscle. Anatomically, two fascicles cannot intersect within the muscle. If any two lines intersect inside the muscle, then the one whose orientation differs most from the median orientation of all the lines is deleted.

**Figure 5:**
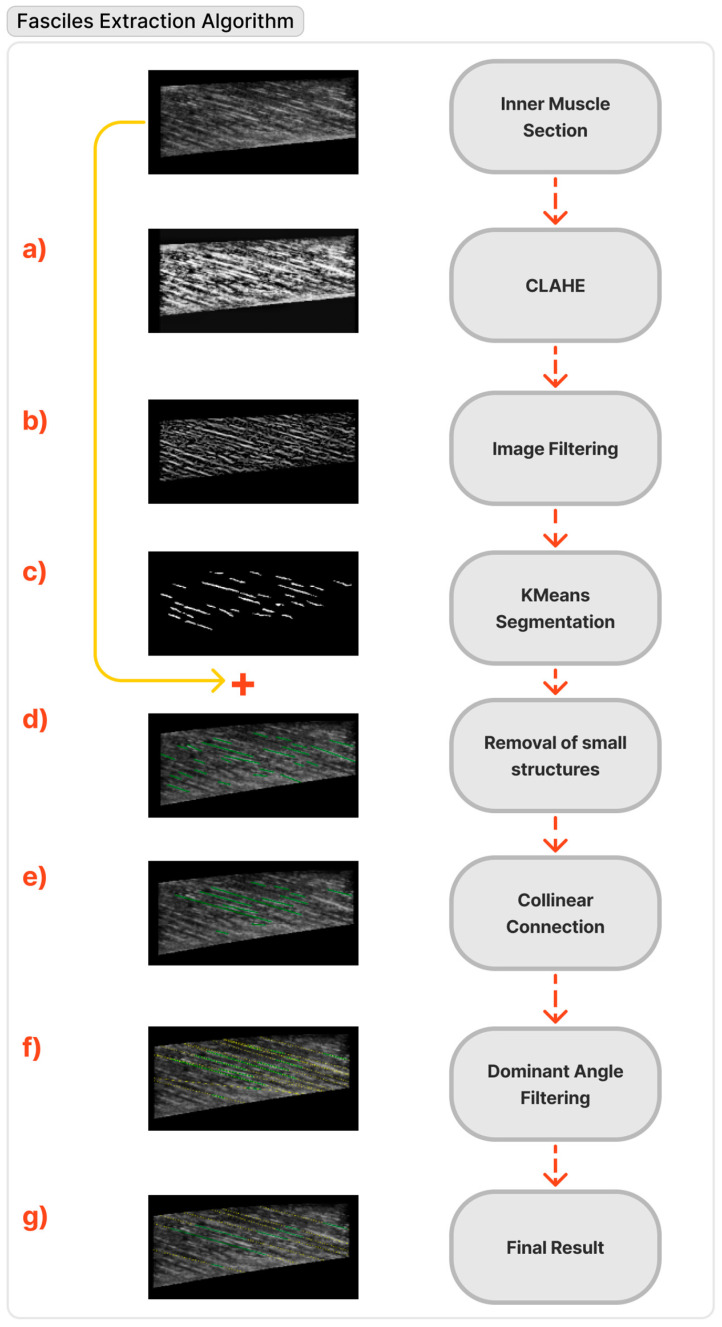
Flow chart of the muscle fascicles extraction pipeline.

The flow chart of the fascicles extraction methodology mentioned above is also depicted in Figure 5.

Some extra notes regarding the implementation of the fascicles extraction algorithm that are worth mentioning are:

- Firstly, for the case of BB muscle, the initial image needs to be horizontally flipped to smoothly apply the procedure mentioned above;
- Secondly, in the case of TA, the two muscle compartments depicted in Figure 1 as (c1) and (c2) have to be isolated (c2 must also be flipped horizontally) to be able to detect the fascicles;
- Lastly, regarding the connection of the algorithm’s collinear segments (step e), at least two of the following criteria must be fulfilled:
  - Their extensions intersect each other within the image;
  - One of the two segments is in the upper left part with respect to the other;
  - Their orientation between the two segments differs less than 5 degrees.

#### 2.3.2. Pennation Angle & Fascicles Length Measurements

**Figure 6:**
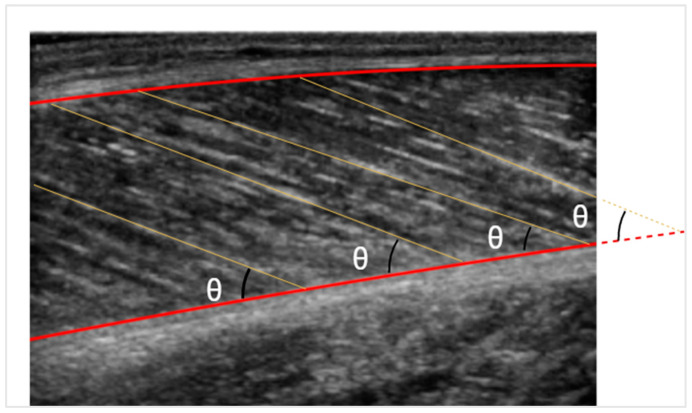
Pennation angle is depicted with *θ*. The fascicle length is illustrated with yellow lines.

After detecting the fascicle lines and defining the muscle boundaries, the pennation angle can be calculated with simple calculus. If, however, the fascicle line intersects the muscle outside the image, as depicted in Figure 6, the deep aponeurosis is extended linearly. On the other hand, the fascicle length is simpler to be measured through trigonometric estimation. Given the muscle thickness and the pennation angle, the fascicle length can be estimated by the formula:

$$L_{F}=d/sin\left(\theta \right)$$

where *d* is the muscle’s thickness, and *θ* is the pennation angle.

Measuring the fascicle’s length this way proved to be the most accurate. However, given our data, the observation was that in most cases, a large portion of the fascicles was not depicted in the images. The hypothesis is that the nominated fascicle portion follows a linear path, which is rarely true in skeletal muscle. Consequently, measuring curved fascicles with trigonometric extrapolation leads to an approximation error.

### 2.4. Evaluation Metrics

Several evaluation metrics were used to assess the proposed method’s performance. First, regarding the image segmentation problem, two well-established indexes were incorporated. Firstly, the Dice Coefficient (Dice Coeff) index, which measures the pixels overlap between two sets of data and secondly, (similarly to the Dice Coeff) the Intersection over Union (IoU) index, which also measures the similarity between two sets of data. Furthermore, the Intra-class Correlation Coefficient (ICC) [32], type ICC (2, 1), was chosen for comparing the reliability of the manual and automated measurements. Finally, the Root Mean Square Error (RMSE) and Bland–Altman plots [33] were also used for a complete evaluation. 

## 3. Results

### 3.1. Muscle Thickness Calculation

In Table 1, a comparison between the Attention UNet and UNet in terms of Dice Coeff and IoU is depicted. The overall performance of the Attention UNet after the post-processing methodology reached over 85% in terms of Dice Coeff, indicating the success of the proposed method. Moreover, the results show that the Attention UNet outperforms the original UNet architecture in all the muscles. Therefore, this is an extra indicator that the Attention UNet can localise the fine-grained details of the muscle tissue, leading to better performance.

In a few cases, Attention UNet could not precisely localise the superficial and deep aponeuroses (in terms of the metrics). Nevertheless, the results are usable for measuring the muscle parameters because most of the accumulated errors and missing information could be fully (or partially) retrieved from the post-processing pipeline. In general, the post-processing algorithm played an important role in filling the missing areas, especially in the aponeuroses’ inner boundaries, which are critical in measuring muscle thickness. Notably, the main source of error was the low contrast image areas which are an inherent disadvantage of the specific image modality since the final quality of the ultrasound images is affected by multiple parameters that cannot always be standardised (e.g., the pressure of the probe, ultrasound machine settings, quantity of gel, etc.). 

Regarding the evaluated muscle sections, the proposed method has achieved the best results in the BB as its images have the most distinct aponeurosis and the majority of images in the evaluated dataset. On the other hand, the TA has slightly underperformed primarily because of its second class, depicted in red in Figure 1, which delineates the central fascia. The central fascia was harder to detect precisely for reasons mostly related to the fact that this category consisted of much fewer samples. The lower scores of the GCM can be attributed to the fact that in many images, the deep aponeurosis is a large structure with no well-defined limits and low-intensity gradients.

Continuing our analysis, Table 2 compares the proposed method and a human operator for calculating muscle thickness. It presents the (mean ± standard deviations) for the muscle thickness measurements and their discrepancy in RMSE and the ICC metrics. 

The evaluation results, which depict the average performance of the whole validation dataset per muscle, have an extremely low RMSE and an ICC close to 1. These results are one more indicator of the reliability of the proposed method for this task. It must also be highlighted that the difference between the human-level performance and the proposed automated process is equal to 0.4 mm, a significant result that can be used for future automation of this clinical task.

Regarding a per muscle analysis, the scores of Table 2 in muscle thickness are consistent with the Attention UNet segmentation and post-processing results. Again, the BB and TA present the best performance of the overall metrics, with GCM following at a close distance. However, even in the case of the GCM, the RMSE is 0.47 mm on average, which is negligible. Lastly, it must be highlighted that the percentage discrepancy between the manual and automatic measurements is 0.82% for the BB, 0.85% for the GCM and 0.5% for the TA. These results show the exceptional performance the proposed method achieves.

**Figure 7:**
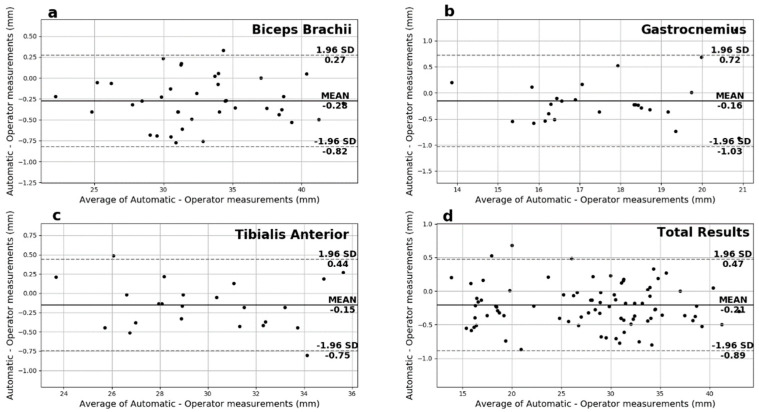
The Bland−Altman plots for the muscle thickness measurements for (**a**) biceps brachii, (**b**) gastrocnemius medialis, (**c**) tibialis anterior and (**d**) overall.

Another useful analysis presented in Figure 7 is the Bland-Altman plots of the muscle thickness measurements. These plots show negligible additive bias and no systematic errors since most differences fall between the 95% limits of agreement. Finally, there are no distinguishable patterns depicted in the plots.

### 3.2. Penation Angle and Fasciles Length Calculation

**Figure 8:**
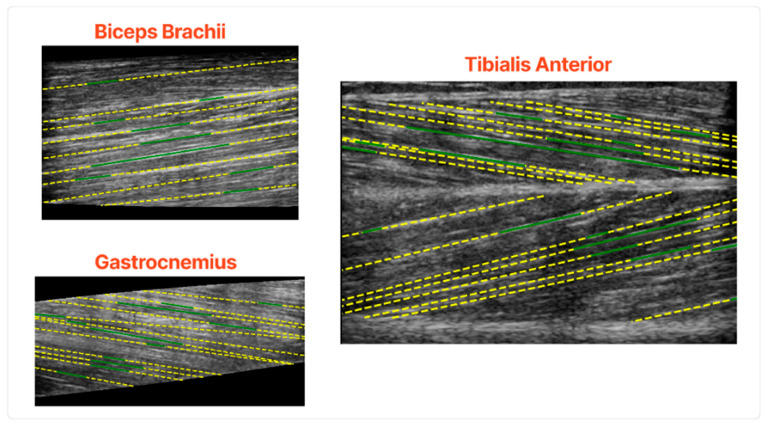
Qualitative results of the fascicles extraction in the validation set. The green lines are the algorithm’s output before the fascicles extended in the whole muscle and the yellow lines after.

In Figure 8, some characteristic results of the fascicles extraction algorithm are depicted. The results show that the proposed pipeline can accurately extract the main fascicles in all the examined muscle sections. Another advantage of the proposed algorithm is that it works in an unsupervised fashion since it does not need exhaustive annotation of the fascicles, a tedious and time-consuming task. 

The fascicles with the highest intensity gradients are the ones which are detected consistently. On the other hand, fascicles of low contrast and not well defined were the ones that the algorithm struggled to delineate. Moreover, the early steps of the algorithm would detect fascicles in multiple small segments, a problem that is tackled in the subsequent steps of the fascicle detection process. 

Continuing the fascicle analysis, Table 3 shows the comparison metrics between the proposed automated process and the human operator for the fascicle length and pennation angle measurements. The results indicate the success of the automated process since, regarding the calculation of the pennation angle, the average difference with the manual measurements equals only 2.22° degrees, a negligible amount. However, regarding the fascicle length measurements, the overall discrepancy was 53.4 mm, with BB contributing more to this error and the other muscles much less. This result can be explained due to the architectural structure of the muscle and its properties. The proposed pipeline showed better performance in the muscles where a significant portion (or all) of the fascicles are located between the superficial and deep aponeuroses. On the contrary, in the BB muscle, the overall accuracy deteriorates since the fascicles are parallel to the aponeuroses. The above happens mostly in errors caused by the trigonometric approximations needed for the calculation.

Regarding a more detailed analysis of Table 3, for the pennation angle, the best RMSE recorded on TA is 1.75°, but both BB (2.09°) and GCM (3.02°) follow in close range. For the fascicle length calculations, the results follow a different trend. In particular, the GCM have an RMSE of 7.65 mm, leading to the best overall results. On the other hand, the TA has a bit higher RMSE of 18.4 mm, and the BB has an RMSE of 87.9 mm, which is noticeably higher for the reasons mentioned earlier.

**Figure 9:**
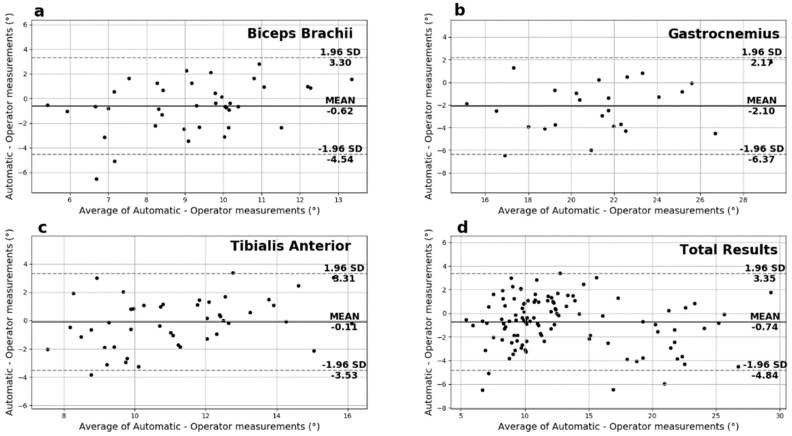
Pennation angle’s Bland−Altman plots for (**a**) biceps brachii, (**b**) gastrocnemius medialis, (**c**) tibialis anterior, and (**d**) overall.

Regarding the Bland–Altman plots of the pennation angle measurements depicted in Figure 9, most differences are between the 95% limits of agreement, thus suggesting that the two measurement methods can be used interchangeably. In addition, these plots show negligible additive bias and no systematic error, thus providing good overall performance.

**Figure 10:**
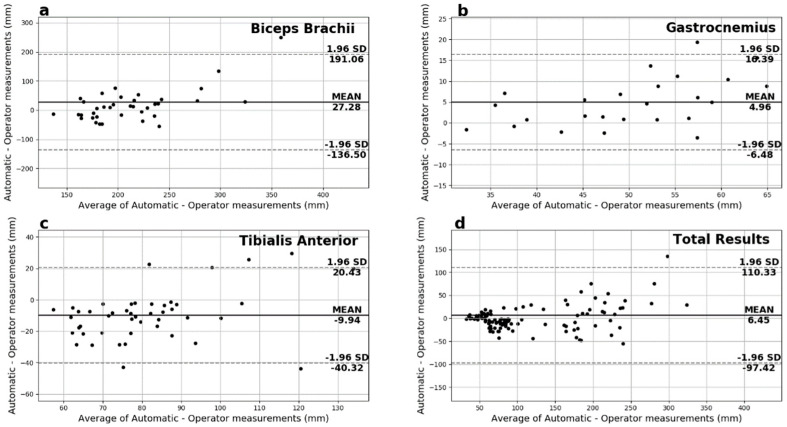
Fascicles Length’s Bland−Altman plots for (**a**) biceps brachii, (**b**) gastrocnemius medialis, (**c**) tibialis anterior, and (**d**) overall.

Finally, on the Bland–Altman plots of the fascicles length measurements depicted in Figure 10, most of the measurements fall between the 95% limits of agreement with no systematic error observed. In particular, GCM presented the smallest additive bias, followed by the TA in close range. However, in the case of the BB, the spread is noticeable and directly comparable to the above results for the fascicles length measurements. That fact reinforces the hypothesis that longer fascicles with a smaller portion shown on the ultrasound images contribute to significant error for the automated system and the experienced operator alike. 

## 4. Discussion

This study initially presented a methodology that successfully automated the calculation of muscle thickness through the automatic delineation of the deep and superficial aponeuroses. Subsequently, the inner muscle area was isolated, and the muscle fascicles were automatically segmented. As the last step, the measurements of pennation angle and fascicles length were extracted. For the realisation of this study, images from three different architectural muscles (BB, GCM and TA) which are very informative for the investigation of neuromuscular disorders and sarcopenia [10,34,35], were selected. Importantly, the analysed ultrasound images were both of high and low contrast between the inner muscle structure and the aponeuroses. 

In comparison with [18], in which the authors used a gradient-based filtering approach to delineate the muscle aponeuroses and extract the muscle thickness, this study presents a solution that is able to generalise better. The improved generalisation ability of the deep learning approaches in relation to classical approaches stems from several reasons, with the most important being the lower sensitivity to noise. In particular, the Attention UNet could learn more complex structures and details from the US images during the training process and through its attention modules. Hence, the aponeuroses could be separated from the background more accurately, even in more challenging scenarios. Another advantage of the pipeline used for extracting the pennation angle and fascicles length measurements is that it works in an unsupervised fashion and does not require extensive image annotation. The annotation process of the muscle fascicles can be tedious and time-consuming, especially in the case of high echogenicity US images which increases the difficulty of their proper localisation. The disadvantage of this method is similar to gradient-based filtering, i.e., the performance can deteriorate in challenging scenarios due to noise. Indeed, further experimentation is needed to accurately estimate the precision of this method in real-world clinical applications.

The limitations of this study primarily emerge from two factors. Firstly, even if the results are encouraging for three different architectural muscles, the human body consists of hundreds of skeletal muscles. Therefore, we cannot be fully confident about the generalisation ability of our algorithms in all of them. Nevertheless, we hypothesise that if these algorithms provide a sufficient number of training examples, our algorithms will be able to adapt and provide adequate performance. Secondly, the ultrasound machines’ variability and settings are also a limitation to consider. All the analyses presented here have been made in images acquired with the same ultrasound machine and image settings. The alteration of this setup can lead to deterioration of the performance in a range that we have not evaluated. However, similarly to the case of the skeletal muscles’ variation, the adaptation of the proposed pipeline can probably be performed effectively, with minimal effort.

Regarding the clinical application of our results, it must be noted that the average discrepancy between the automatic and manual measurements was equal to 0.72% for the muscle thickness calculation. Comparing this result with the precision needed in clinical practice indicates that our results are not just acceptable but also reliable. In particular, it is presented in [34] that the accuracy required for diagnostic purposes must be in the range of 5–10% (e.g., in the case of muscle hypotrophy) or even above 10% in the case of diagnosing muscle atrophy or sarcopenia [10]. Another use of this study can be integrating the presented methods in a broader scheme of automatisation of ultrasound measurements in clinical routine. In [36], the authors presented a procedure to automatically collect ultrasound images of lateral abdominal muscles without the need to visually inspect the position of the transversus abdominis (TrA) muscle that usually controls this process. Our proposed methodology can be used as a complementary tool to this method for identifying and extracting muscle parameters automatically from these US images.

This study aims to improve already existing CAD systems for calculating muscle parameters through highly advanced deep learning techniques. Our results indicate that such an objective is feasible since our method achieves human-level performance, a challenging task for the existing software solutions. Regarding the automatic delineation of the deep and superficial aponeurosis and the muscle thickness calculation, all three muscles exhibited reliable results, with the BB muscle images having the lowest RMSE and highest ICC (2,1). This result is consistent with the properties of the specific muscle since the deep and superficial aponeuroses in this muscle were slim with high contrast linear structures, making them easier to detect. Concerning the pennation angle calculations, the average error was only 2.22°, a result that portrays the robustness of the proposed algorithm. Finally, the GCM images had the lowest RMSE (7.65 mm) for the fascicle length calculation, and the BB had the higher (87.9 mm). This behaviour is mainly credited to images of the GCM since most intersections between the fascicles and aponeurosis are depicted inside the muscle section. In contrast, in the case of the BB muscle, most images depicted only a small portion of the fascicle, so the rest was estimated through trigonometry, resulting in higher error. 

The time needed for the aponeurosis segmentation, muscle thickness calculation, fascicle detection, pennation angle and fascicle length calculation was 2.5 s, which certainly does not induce a significant time burden in the clinical task. The benchmarking was performed on a system with an Intel Core i5-8400 CPU and GeForce RTX 2070 ARMOR 8GB GPU. The fascicle segmentation was the lengthiest of these processes, with about 1.5 s duration. That is mainly because this process runs on the CPU and does not take advantage of the more efficient GPU. We plan to optimise the proposed algorithms in the future to increase the overall runtime performance.

In future work, we plan to assess the proposed methodology in ultrasound images acquired at different anatomical sites in the human body. Significantly, these measurements will be acquired from subjects who present US images with a normal echogenicity and patients with sarcopenia, who have a loss of muscle architecture that leads to increased echogenicity and lower contrast muscle structures. 

## Figures and Tables

**Table 1:** Results of UNet and Attention UNet in terms of Dice Coeff | IoU.

Muscles	UNet	Attention UNet
BB	0.86 | 0.77	0.88 | 0.79
GCM	0.75 | 0.61	0.81 | 0.68
TA	0.78 | 0.65	0.85 | 0.73
Total	0.77 | 0.65	0.85 | 0.74

**Table 2:** Muscle thickness measurements evaluation results.

Muscle	Operator (mm)	Automatic Method (mm)	RMSE (mm)	ICC (2,1)
BB	32.93 ± 4.72	32.66 ± 4.72	0.39	0.99
GCM	17.70 ± 1.76	17.55 ± 1.85	0.47	0.97
TA	29.95 ± 3.28	29.80 ± 3.22	0.34	0.99
Total	-	-	0.40	0.99

**Table 3:** Comparative RMSE results of the pennation angle and fascicle length measurements. The fascicle length is measured in mm and the pennation angle in degrees.

Muscle	Pennation Angle (°)	Fascicles Length (mm)
BB	2.09	87.9
GCM	3.02	7.65
TA	1.75	18.4
Total	2.22	53.4

## Data Availability

Not applicable.
